# Phototaxis of Cyanobacteria under Complex Light Environments

**DOI:** 10.1128/mBio.00498-17

**Published:** 2017-04-11

**Authors:** Minsu Kim

**Affiliations:** Emory University, Atlanta, Georgia, USA

**Keywords:** cell motility, cyanobacteria, phototaxis

## Abstract

Photosynthetic bacteria are capable of producing their own food via photosynthesis. Unsurprisingly, they evolved the ability to move toward better light conditions (i.e., phototaxis). In a recent article in *mBio*, Chau et al. tuned the wavelength, flux, direction, and timing of light input and characterized the motility of the unicellular cyanobacterium *Synechocystis* sp. strain PCC6803 (R. M. W. Chau, D. Bhaya, and K. C. Huang, mBio 8:e02330-16, 2017, https://doi.org/10.1128/mBio.02330-16). The results revealed an intricate dependence of the motility on various light inputs, laying the fundamental groundwork toward understanding phototaxis under complex and dynamic light environments.

## COMMENTARY

The movement of heterotrophic bacteria under a nutrient gradient is a well-understood process. Heterotrophic bacteria cannot fix carbon from inorganic sources and have to use organic carbon for their growth. These bacteria do not wait in one place for organic molecules to diffuse toward them; rather, they move toward the sources of such molecules. Since the observation, first published more than 100 years ago, that bacteria swim into capillaries filled with meat extract but escape from capillaries filled with poison (1–3), extensive studies have characterized chemotaxis in heterotrophic bacteria. Now, much is known about how these bacteria regulate their motility in response to different chemical signals and chemical compositions of their environment.

Phototrophic bacteria, i.e., those that can synthesize the organic compounds they need directly by using the energy from light, face a similar need to move. These bacteria require light to convert water and carbon dioxide to carbohydrates and oxygen during oxygenic photosynthesis. Like their heterotrophic counterparts and their responses to chemical signals, phototropic bacteria have evolved the ability to sense light and move toward better light conditions via a process called phototaxis. Unlike chemotaxis, our understanding of bacterial phototaxis is limited. For example, when multiple light sources are presented from different directions, in which direction do the bacteria move? Not all wavelengths of light are equally beneficial, as some wavelengths (blue or ultraviolent) can damage DNA and other cellular components. Do the cells respond to light of different wavelengths differently? In their natural habitats, they experience various light intensities and wavelengths. How quickly can they respond to such changes in light inputs?

In a recent study published in *mBio*, Chau et al. addressed these fundamental questions about bacterial phototaxis by subjecting unicellular the cyanobacterium *Synechocystis* sp. strain PCC 6803 to well-controlled light environments (4). Cyanobacteria are the only known form of bacteria that perform oxygenic photosynthesis. *Synechocystis* sp. PCC 6803 is a model cyanobacterium for studies of phototaxis and photosynthesis, because it is the first phototrophic organism that has been fully sequenced (5), and a strategy to delete specific genes is available. This bacterium measures light intensity and color by using a range of photoreceptors. It cannot swim, but it can crawl across surfaces by using type IV pili; it crawls by extending, adhering, and retracting the pili. Therefore, experiments to measure phototaxis of cyanobacteria are typically performed on an agarose surface. Previous studies have shown that in the dark, cells remain motile but their motion is unbiased (6). In the presence of a white light source, the community of cells forms finger-like projections toward the light source (7).

In their study, Chau et al. tuned the wavelength, flux, direction, and timing of light input and characterized the finger-like projections of the bacterial community as well as the motility of cells within the community. Their findings were interesting: cells moved toward green or red light sources, not because those lights increased the speed of individual cells but because they caused motility bias. The bias was greater with higher light flux. When cells were exposed to multiple light sources, they did not simply react to the dominant one. Rather, they integrated information from multiple light inputs and had a coordinated phototactic response. For example, when two light sources were placed perpendicular to each other, the bacteria moved along the vector sum of the two light paths. When two light sources were placed in opposing directions, signals counteracted each other, resulting in a lack of phototaxis of the community. Importantly, this lack of phototaxis was not because the motility of cells was abolished (individual cells within the community maintained nonzero speed). Rather, it was because there was no bias in the motion of individual cells within the community.

Furthermore, the motility strongly depended on the wavelength of light, as blue light did not induce phototaxis. Single-cell-level measurements showed that blue light completely inhibits the motility of cells. When green light was simultaneously presented with blue light, the inhibition was relieved. When a green light was presented alone initially and turned off, the bias in the motility was lost quickly (within ~10 min) and reemerged quickly (within ~10 min) when the green light was turned back on. When the green light, rather than being switched off, was switched to blue light, the motility was lost quickly. However, when the blue light was switched back to green light, it took ~40 min for the motility to reemerge. Therefore, recovery from the loss of the motility takes significantly longer than recovery from the loss of the bias in the motility. Furthermore, the results indicated that phototaxis strongly depends on the wavelength of light, a point missed in previous studies, which frequently used a white light source.

Although this study did not directly address mechanisms of light sensing and motility regulation, its findings suggest interesting possibilities. First, the results of this study showed that when motility bias increases in a light flux-dependent manner, the speed of cells is maintained. It is thus unlikely that pilus activity increases with stronger light flux, but rather, the ratio of pulling toward versus away from the light determines the motility. Second, the intensity dependence of the competition between green light and blue light inputs suggests that none of the photoreceptors has a dominant effect. Third, phototactic responses upon a light shift suggest that timescales of photoreceptor activation/deactivation. These results can serve as constraints for mechanisms of light sensing and motility regulation, guiding future mechanistic studies.

Lastly, the ability of *Synechocystis* cells to “choose” the vector sum of different light directions is striking. These cells are spherical, have an ~1 µm radius, and are not much larger than the wavelength of light used for the study (the wavelength of green light is approximately 0.5 µm). How do these tiny cells detect the directions of light sources? A recent article suggested that the cells may act as spherical microlenses, which focus the light at the rear periphery of the cell, and the focused light induces cell motion in the opposite direction (8). If so, *Synechocystis* sp. would be the smallest organism to use optical lensing to “see” light.

Heterotrophic bacteria evolved a sophisticated strategy to navigate in complex chemical environments. The Chau et al. study clearly demonstrates that, similarly, phototactic bacteria evolved a sophisticated strategy to navigate in complex light environments. Such a phototactic strategy requires intricate sensory and regulatory mechanisms. Future studies of such mechanisms may reveal novel principles of light sensing and motility regulation by phototactic bacteria. How will these principles underlying phototaxis compare with those underlying chemotaxis? Time will tell.
